# Supplementary material for: Analysis of pulsed cisplatin signalling dynamics identifies effectors of resistance in lung adenocarcinoma
Source: eLife. 2020 Jun 9;9:e53367. doi: 10.7554/eLife.53367 (PMC7282820; doi:10.7554/eLife.53367)
Supplement: Supplementary file 1. [file elife-53367-supp1.docx]

**Supplementary File 1: Protein analytes previously implicated in the response to cisplatin.**

| **Protein** | **Rationale** | **Refs** |
| --- | --- | --- |
| **p53** | P53 is known to be an integral component of the signalling response to DNA damage. | (40) |
| **Chk1** | ATR-Chk1 signalling occurs in response to single-strand DNA breaks and plays a critical role in DNA damage and replication checkpoints. | (41) |
| **Chk2** | ATM-Chk2 signalling occurs in response to double-strand DNA breaks and prompts DNA repair through homologous recombination. | (41) |
| **H2AX** | H2A.X is a core component of the DNA damage response. Phosphorylation at Serine 139, termed γH2A.X, is a well-established biomarker for DNA double-strand breaks. | (42) |
| **MDM2** | A component of the p53 signalling pathway. Negatively regulates p53 activity and has been shown to contribute to cisplatin sensitivity in testicular cancer cells. | (43) |
| **p21** | A transcription target of p53 signalling. Decreased expression of p21 may indicate sensitivity toward cisplatin treatment. | (43) |
| **JNK** | A protein kinase with a well characterised involvement in the stress response. Inhibition of JNK has been shown to sensitise platinum-resistant NSCLC cells to cisplatin treatment | (44) (45) |
| **p38** | A protein kinase with a well characterised involvement in the stress response. p38 activation has been previously demonstrated to correlate with a cisplatin resistant phenotype. | (27) (30) |
| **ERK1/2** | The ERK1/2 pathway is a major cell survival pathway that is frequently upregulated in cancer cells. Inhibition of this pathway has been shown to sensitise lung cancer cells to cisplatin treatment. | (46) |
| **MCL-1** | An anti-apoptotic member of the Bcl-2 family. Knockdown of MCL-1 has been shown to increase sensitivity of lung cancer cells towards platinum. | (12) |
| **MCL-1/BAK dimer** | BAK is a pro-apoptotic Bcl-2 family protein that is neutralised when forming a dimer with MCL-1. | (47) |
| **Cleaved caspase 3** | Caspase 3 cleavage is a quintessential feature of apoptosis. | (48) |
