# Supplementary material for: Analysis of pulsed cisplatin signalling dynamics identifies effectors of resistance in lung adenocarcinoma
Source: eLife. 2020 Jun 9;9:e53367. doi: 10.7554/eLife.53367 (PMC7282820; doi:10.7554/eLife.53367)
Supplement: Supplementary file 2. [file elife-53367-supp2.docx]

**Supplementary File 2: Mutation status of the lung adenocarcinoma cell panel.**

| **Cell line** | **TP53** | **RAS** | **EGFR** | **CDKN2A** |
| --- | --- | --- | --- | --- |
| **A549** | WT | KRAS G12S | WT | Del (Hom) |
| **SW-1573** | WT | KRAS G12C | WT | Del (Hom) |
| **NCI-H1573** | R248L (Hom) | KRAS G12A | WT | WT |
| **NCI-H1975** | R273H (Hom) | WT | T790M, L858R | WT |
| **NCI-H358** | Del (Hom) | KRAS G12C | WT | WT |
| **NCI-H1299** | Del (Hom) | NRAS Q61K | WT | WT |
