# Supplementary material for: Analysis of pulsed cisplatin signalling dynamics identifies effectors of resistance in lung adenocarcinoma
Source: eLife. 2020 Jun 9;9:e53367. doi: 10.7554/eLife.53367 (PMC7282820; doi:10.7554/eLife.53367)
Supplement: Supplementary file 3. [file elife-53367-supp3.docx]

**Supplementary File 3: Summary of the analytes used for multiplex signalling analysis.**

| **Pathway** | **Analytes** |
| --- | --- |
| DNA Damage Response | ATR (total), Chk1 (Ser345), Chk2 (Thr68), P53 (total), P53(Ser15), P53 (Ser46), MDM2 (total), P21 (total), γH2AX (Ser139), cleaved PARP |
| AKT/mTOR Signalling | IRS-1 (Ser636/639), Akt (Ser473), BAD (Ser136), BAD (Ser112), Bcl-2 (Ser70), GSK-3α/β (Ser21/9), S6 ribosomal protein (Ser235/236), PTEN (Ser380), mTOR (Ser2448), P70S6K (Thr389) |
| MAPK Signalling | MEK1 (Ser217/221), ERK1/2 (Thr202/Tyr204), P90RSK (Ser380), P38 (Thr180/Tyr182), HSP27 (Ser78), JNK (Thr183/Tyr185), c-Jun (Ser63), ATF2 (Thr71), STAT1 (Tyr701), STAT3 (Ser727) |
| TGFβ Signalling | TGFβRII (total), Smad2 (Ser465/467), Smad3 (Ser423/425), Smad4 (total) |
| NF-κB Signalling | NF-κB (Ser536), IκBα (Ser32/36) |
| Apoptosis Regulators | BAD (total), Bcl-xL (total), BIM (total), MCL-1 (total), Bcl-xL/BAK dimer (total), MCL-1/BAK dimer (total), BAX/Bcl-2 dimer (total), Survivin (total) |
| Apoptosis Effectors | Active Caspase-8, Active Caspase-9, Active Caspase-3 |
