# Supplementary material for: Analysis of pulsed cisplatin signalling dynamics identifies effectors of resistance in lung adenocarcinoma
Source: eLife. 2020 Jun 9;9:e53367. doi: 10.7554/eLife.53367 (PMC7282820; doi:10.7554/eLife.53367)
Supplement: Supplementary file 4. [file elife-53367-supp4.docx]

**Supplementary File 4: Characteristics of the patient cohort used to generate immuno-histochemical data.**

**n %**

***Total Samples***  52

***Histology*** Adenocarcinoma 50 96.2%

Large cell favouring adenocarcinoma 1 1.9%

Mucinous adenocarcinoma 1 1.9%

***Stage***  IV 36 69.3%

IIIA 8 15.4%

IIIB 5 9.6%

III 1 1.9%

IIB 1 1.9%

II 1 1.9%

***Chemotherapy*** Carboplatin/Gemcitabine 28 53.8%

Carboplatin/Pemetrexed 3 5.8%

Carboplatin/Pemetrexed +/- Pembrolizumab 4 7.7%

Carboplatin/Taxol + Bevacizumab 2 3.8%

Concurrent Carboplatin/Taxol 11 21.2%

Concurrent Cisplatin/Etoposide 3 5.8%

Concurrent Carboplatin + Unknown 1 1.9%
