# Supplementary material for: Analysis of pulsed cisplatin signalling dynamics identifies effectors of resistance in lung adenocarcinoma
Source: eLife. 2020 Jun 9;9:e53367. doi: 10.7554/eLife.53367 (PMC7282820; doi:10.7554/eLife.53367)
Supplement: Supplementary file 5. [file elife-53367-supp5.docx]

**Supplementary File 5: Mutation status of second lung adenocarcinoma cell panel.**

| **Cell line** | **TP53** | **RAS** | **EGFR** | **CDKN2A** |
| --- | --- | --- | --- | --- |
| **A-427** | WT | KRAS G12D | WT | Del (Hom) |
| **NCI-H2122** | Q236L, C176F | KRAS G12C | WT | WT |
| **NCI-H1792** | WT | KRAS G12C | WT | W110* |
| **NCI-H1944** | WT | KRAS G13C | WT | Del(Hom) |
| **NCI-H2009** | R273L | KRAS G12A | WT | WT |
| **NCI-H727** | Q165insYKQ | KRAS G12V | WT | WT |
| **NCI-H292** | WT | WT | WT | Del(Hom) |
| **NCI-H23** | M264I (Hom) | KRAS G12C | WT | WT |
